# Broken Rotor Bar Detection Based on Steady-State Stray Flux Signals Using Triaxial Sensor with Random Positioning

**DOI:** 10.3390/s24103080

**Published:** 2024-05-12

**Authors:** Marko Zubčić, Ivan Pavić, Petar Matić, Adam Polak

**Affiliations:** 1 Faculty of Maritime Studies, University of Split, Ruđera Boškovića 37, 21000 Split, Croatia; mzubcic@pfst.hr (M.Z.); pmatic@pfst.hr (P.M.); 2 Faculty of Mechanical and Electrical Engineering Polish Naval Academy, ul. Smidowicza 69, 81-127 Gdynia, Poland; a.polak@amw.gdynia.pl

**Keywords:** squirrel cage induction motor, broken rotor bar detection, sensor random position, parametric test, non-parametric test

## Abstract

This paper investigates the detection of broken rotor bar in squirrel cage induction motors using a novel approach of randomly positioning a triaxial sensor over the motor surface. This study is conducted on two motors under laboratory conditions, where one motor is kept in a healthy state, and the other is subjected to a broken rotor bar (BRB) fault. The induced electromotive force of the triaxial coils, recorded over ten days with 100 measurements per day, is statistically analyzed. Normality tests and graphical interpretation methods are used to evaluate the data distribution. Parametric and non-parametric approaches are used to analyze the data. Both approaches show that the measurement method is valid and consistent over time and statistically distinguishes healthy motors from those with BRB defects when a reference or threshold value is specified. While the comparison between healthy motors shows a discrepancy, the quantitative analysis shows a smaller estimated difference in mean values between healthy motors than comparing healthy and BRB motors.

## 1. Introduction

The induction motor (IM) is an alternating current (AC) electrical machine used to drive various industrial utility components such as compressors, pumps, fans, elevators, cranes, etc. There are two types of IMs: wound rotor induction motors (WRIMs) and squirrel cage induction motors (SCIMs). As stated in [1], SCIMs account for ≈87% of the total AC motor population in the industry.

Faults in SCIMs are divided into two categories: electrical and mechanical faults. Electrical faults are further divided into stator (turn-to-turn, coil-to-coil, phase-to-phase, phase-to-ground, and open circuit), rotor (broken rotor bar and broken end ring), and power supply faults (phase imbalance and single phasing). Mechanical faults are divided into stator (frame vibrations), rotor (unbalanced, bent rotor, static, dynamic, and mixed eccentricity), and bearing faults (outer ring, inner ring, rolling elements, and loss of lubricant) [2,3].

**Figure 1:**
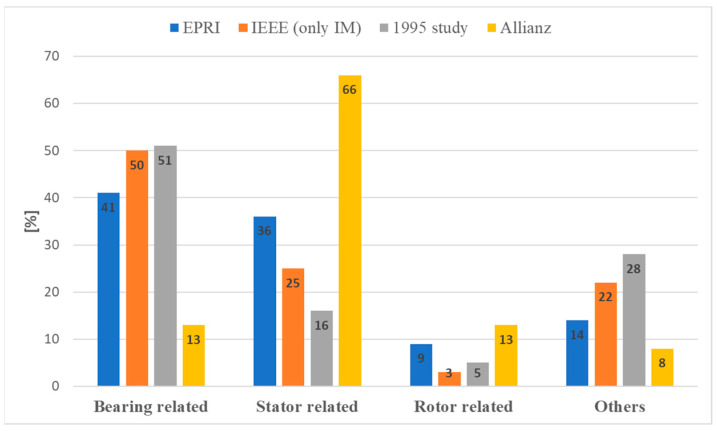
Squirrel cage induction motor fault distribution.

The distribution of the fault types analyzed in various studies is shown in Figure 1. The studies shown in Figure 1 are the Electric Power Research Institute (EPRI) study, the Motor Reliability Working Group (MRWG) study of the Institute of Electrical and Electronics Engineers (IEEE), the 1995 study, and the Allianz study [4]. The EPRI study deals with SCIMs, WRIMs, and synchronous motors (100 hp and above at low voltage levels 460 V and 575 V, and medium voltage motors 2.3 kV, 4 kV, 6.6 kV, and 13.2 kV) [5]. The 1995 study [6] covers SCIMs of 10 kW and above. The IEEE MRWG study covers asynchronous, synchronous, wound-rotor, and DC motors over 200 hp that are not older than 15 years [7,8]. The Alliance study covers medium-voltage motors with high power [9].

The methods for detecting broken rotor bars are divided into model-based and signal-processing methods. The model-based methods are further divided into methods based on resistance estimation (they use the deviation of the estimated rotor resistance from the known value as a fault indicator), methods based on estimation of other parameters (estimation of stator current, rotor flux, rotor speed, etc.), and methods based on a digital twin (they automatically measure and estimate motor parameters and variables based on online data). The methods based on signal processing are divided into the time domain (decomposition products of currents and voltages, differential measurement of the air-gap magnetic field [10], etc.), the frequency domain (fast Fourier transform), and the time–frequency domain (short-time Fourier transform, Chriplet transform, Wigner–Ville distribution, Hilbert–Huang transform, continuous wavelet transform, discrete wavelet transform, etc.) [11,12].

Magnetic flux fault detection is based on the direct or indirect measurement of the magnetic flux or magnetic flux density. Depending on the location of the measurement, there is an external or internal detection method. Internal magnetic flux or air gap flux detection is based on the measurement of the magnetic flux or magnetic flux density in the air gap of the electrical machine. Although air-gap flux detection was developed in the 1970s to detect faults in the stator winding of synchronous generators, it is still an active field of research [13]. This type of fault detection is considered invasive as it requires access to the air gap or stator slots. This means that the work process must be stopped, the electrical machine dismantled, and the sensor carefully positioned. Proper installation of air gap sensors can be carried out during an overhaul or manufacturing process.

The external magnetic flux or stray flux is the magnetic flux that radiates into the environment of the electrical machine. Although the magnitude of the stray flux is much weaker than the air-gap flux, this physical quantity reflects the anomalies (asymmetries) of the total magnetic field in the electrical machine [14]. The oldest paper on monitoring stray flux available on the Web of Science dates back to 1971 [15]. In the paper, the authors investigated an unbalanced supply. The aim was to detect certain frequency components in the spectrum of the coil voltage and use this information to activate the protective device that disconnects the motor from the mains in the event of an unbalanced supply. The air-core coil was placed on the non-drive side of the motor.

The motivation for extracting information about the motor state with random positioning of the sensor is based on the review articles [12,16,17,18], the literature review presented in the next section of this paper, and the assumption that the human factor plays a role in the monitoring process. From the review articles and the literature review, it can be seen that the research papers study BRB faults with a stationary position of the sensor, i.e., the sensor is placed at a fixed position. This study is conducted under the assumption that the operator has his own choice regarding the sensor position, e.g. because there are no guidelines or instructions for the sensor position. Because of this freedom of choice, the authors refer to this approach as random.

Two objectives of this paper are to validate the measurement method and to investigate BRB detection in a steady state using the magnetic stray flux method with random positioning of the sensor over the surface of the motor. The validation of the measurement method requires that the information about the state of the motor (healthy or BRB) is time-independent, i.e., if the measurement is performed with random positioning at different times for a given motor then all measurements must not show a statistically significant difference if the motor has not changed its state. If the motor changes its state from healthy to BRB at a certain point in time, the measurements before and after must show a statistically significant difference. Appropriate statistical tests based on the distribution of measurement data are used for the validation and detection of BRB.

The investigations carried out by the authors take place under laboratory conditions.

## 2. Literature Review

**Figure 2:**
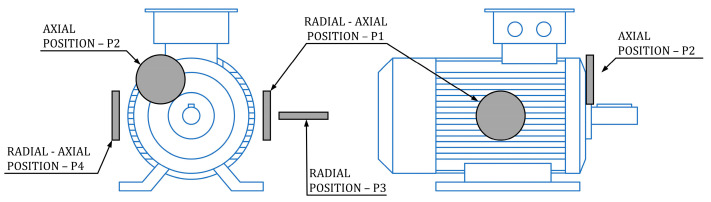
Different fixed sensor positions.

In this section, a brief description of papers related to SCIM broken bar detection using stray magnetic flux is given. A common feature of all stated papers is the fixed positions of the sensor. The fixed position of the sensor refers to a situation where the sensor is once positioned for measuring, and it is not translated or rotated to a new position for a new measurement during the overall measurement process. The information about the motor state (healthy/BRB) is extracted and processed from the same position of the sensor. There are three standard positions of the sensor: axial, radial, and radial–axial. These three positions are a common feature of all the following articles. Figure 2 shows the standard positions of the sensors and serves as a reference for all articles in this section.

The articles presented in this section emphasize the fixed sensor position and serve as a reference point regarding used methods in this field of research. Together with review articles [12,16,17,18], they provide insight that random sensor positioning during the measurement process and its potential for motor state information extraction have not been investigated.

In [19], the authors used a laboratory-made air-core coil as a sensor. The analyzed signal was the EMF of the air coil, and the faults were analyzed when the motor was started. Tests were also performed on two induction motors and for three positions of the sensor: radial, axial, and radial–axial. The short-time Fourier Transform (STFT) was applied to transient EMF signals to show the evolution of the fault harmonics. A rotor fault indicator based on the discrete wavelet transform (DWT) was introduced. The authors conclude that misalignment and BRB can be detected with the STFT of the air coil signal. The current analysis shows that the BRB fault can mask the misalignment on the time-frequency map; the rotor fault indicator shows a significant difference compared to the healthy motor; different sensor positions give different rotor fault indicator values, and the largest difference to the healthy condition is reported for the axial position.

In [20], the detection of two adjacent broken bars, two broken bars within half a pole pitch, and two broken bars with one pole pitch is investigated. The sensor used was an air-core coil. The signal was analyzed by first performing an STFT with the motor in a steady state and extracting the four frequency trajectories. After the trajectories were extracted, the Fast Fourier Transform (FFT) was applied. The fault detection is based on the observation of the missing frequency component in the frequency spectrum of the healthy motor compared to the fault scenarios. The presented method detects broken bars, adjacent and non-adjacent, with half pole pitch and with one pole pitch. This method can be used as a complement to Motor Current Signal Analysis (MCSA), as the latter provides a false negative misdiagnosis for broken bars with half pole pitch and with one pole pitch.

In [21], the authors propose an algorithm based on a sorted spectrum subtraction of healthy and faulty (different spatial combinations of two BRBs) motor states. The algorithm was tested for the following combinations of broken bars: 1-2 (adjacent), 1-3, 1-4, 1-5, and 1-6. To quantify the distinction between healthy and faulty states, a fault indicator based on autocovariance was introduced. For comparison, the authors calculated the ratios of the indicators by dividing each indicator for a faulty condition by the indicator for a healthy motor. The calculation was performed for axial and radial–axial coil position and the results show quantitative differences and different sensitivity depending on the position of the sensor. It is reported that the radial–axial position is more sensitive than the axial position.

In [22], the detection of a broken bar is investigated using two indicators. The first is based on the frequency domain and is calculated as the sum of the average of the absolute values of the bispectrum; the second indicator is based on the time domain and is calculated as the squared value of the median of the autocorrelation function. The tests were carried out during start-up and in a steady state. An air-core coil was used as the sensor. The measurements with the coil were carried out in four positions: radial, axial, and radial–axial (P1 and P4). When analyzing the frequency and time indicators for the steady state, it is noticeable that all faulty values are lower than in the healthy state, with the exception of sensor position P4 in both cases. The faulty value of the frequency indicator for the start-up regime has a higher value than the value for the healthy state. The values of the time indicator for the start-up process show that all faulty values are lower than the values in the healthy state, with the exception of position P2.

In [23], the authors investigate the two-stage time-frequency analysis for detecting broken bars in a steady state. The authors use STFT with Kaiser–Bessel window function. The expression for the minimum window length is derived in the paper. The two-stage analysis was used to study the 5th and 7th harmonics and their sidebands. The conclusion of the paper is that broken rotor bars can be detected with the SFTF at a steady state and with the minimum window length derived in the paper.

In [24], the authors investigated the influence of the axial air channels of the rotor on the detection of broken rotor bars. The motivation for this research was the misdiagnosis of the MCSA method. If the number of axial channels is equal to the number of poles, the MCSA can generate a false positive or false negative alarm. The motor was tested with 0, 1, and 2 (adjacent) broken bars. The sensor used was an air-core coil placed in a radial position. The stator current and the radial flux (EMF of the coil) were measured for comparison. FFT was used for signal analysis, and the spectral component (1-2s)f was analyzed. The work showed that the detection of rotor fracture is independent of the presence of an axial channel when magnetic stray flux analysis is applied.

In [25], the authors propose a method for automatic detection of a BRB based on a multiple signal classification algorithm (MUSIC) and an artificial neural network (ANN). An air coil was used as the sensor for the measurement of magnetic stray flux. The success rate achieved with the proposed algorithm in detecting BRB faults shows the potential for autonomous fault detection based on stray magnetic flux.

In [26], the authors proposed a method for the automatic detection of broken rotor bars, misalignment, and combinations: BRB + misalignment. The proposed method is based on STFT, statistical parameters, feature extraction, linear discriminant analysis (LDA), dimensionality reduction, and feed-forward neural network (FFNN). The sensor used was a triaxial stray flux sensor. The triaxial sensor consists of three Hall sensors whose axes are perpendicular to each other, and which are all installed on one circuit board. They are arranged so that one sensor detects the axial flux, the second the radial flux, and the third the radial–axial flux. The proposed algorithm can automatically detect the healthy state, misalignment, and Misal. + 1 BRB and Misal. + 2 BRB. For all states, the authors report that the effectiveness is more than 95%.

In [27], the authors investigated the detection of a BRB by monitoring the rotor rotational frequency and the supply frequency sidebands. The authors investigated one BRB, two BRBs (adjacent), and two non-adjacent BRBs separated by 90° (electrically). The faults were investigated using three methods: MCSA, the internal magnetic flux (air gap), and stray magnetic flux. For the stray magnetic flux method, an air-core coil in the radial–axial position was used. The magnetic stray flux in the steady state and the *fs* − *fr* frequency component, where *fs* is supply and *fr* is the rotor rotational frequency, can be used to identify all faulty conditions. The FFT and STFT analysis of the *fs* − *fr* frequency component showed that the internal magnetic flux can detect all faulty states. The authors also showed in experiments that the *fs* − *fr* frequency component of the internal and external magnetic flux does not respond to an unbalanced load.

In [28], the authors investigated the effects of a BRB on the mechanical frequencies. An air coil with a square cross-section mounted on the fan cover of the motor in a radial–axial position (P1) is used as a sensor. The motor current was measured for comparison. The measurements were carried out at the rated load of the motor. The analysis of the mechanical frequencies in the steady state to detect rotor faults shows diagnostic potential.

In [29], the authors proposed the fifth harmonic of the rotor rotational frequency as an indicator of rotor faults in induction motors: *fs* + 5*fr*. The fifth harmonic was chosen because it does not contain sidebands that cause false indications and has a low sensitivity. Stray flux analysis of the *fs* + 5*fr* component shows that all faulty states (one BRB and two BRB—adjacent and non-adjacent) are detectable compared to the spectrum of the healthy state and the defined threshold: −66.4 dB. The proposed indicator is immune to the presence of an axial air channel. The indicator is not affected by an unbalanced load and misalignment.

A diagnostic study conducted under real conditions is described in [30]. The diagnosis took place in a pumping station. There was no prior knowledge of the parameters of the mechanical system. The diagnosis was performed with MCSA, stray flux, and vibration analysis with the motors in a steady state. The flux was monitored with two air coils in the radial–axial position (P1). The work shows that the stray flux is not sensitive to mechanical faults originating from the load. A second method was required to localize the fault (the pump system studied is a complex electromechanical system—vertically mounted SCIM, impeller, 15 m shaft, and 6 bearings). In this example, one method alone cannot provide complete screening (fault detection and fault localization).

In [31], fault detection with stray flux in motors with soft starting is investigated. Four soft starters were used. The signals of the air coils were determined for the radial, axial, and radial–axial positions in the steady state and start-up condition. The steady-state signals were analyzed with FFT, and the start-up signals with STFT. The variables in the tests were the time setting and the initial torque/current setting. The faults investigated were as follows: one BRB without load and with load and two BRBs (adjacent) without load and with load. The authors proposed the following fault indicator: the highest value of the sf component from the STFT analysis. Broken rotor bars can be detected when the motor is operated with a soft starter. The transient stray flux, together with the steady-state stray flux, provides reliable information about the rotor fault.

In [32], an automatic diagnostic system based on stray flux and current data is investigated. The diagnostic system consists of the following steps: (1) stray flux and stator current data at start-up, (2) STFT application, (3) division of the STFT map into regions and calculation of a proposed indicator for each region, (4) classification of the condition (healthy, one BRB, two BRB, misalignment) by FFNN, and (5) final diagnosis for the end user via a user interface. The sensor used for stray flux was a triaxial sensor consisting of three Hall sensors on a board arranged perpendicular to each other. The results show 100% effectiveness in detecting two BRBs, 100% effectiveness in detecting one BRB, 95% effectiveness in detecting misalignment, and 100% effectiveness in detecting a healthy motor.

In [33], the possibility of monitoring the tool condition in a CNC machine is investigated. The monitored object was a cutting tool. The idea was to monitor the wear of the cutting tool indirectly by monitoring the stray flux of the spindle motor. The proposed method for tool wear estimation consists of the following steps: (1) Data acquisition from triaxial sensors (3 perpendicular Hall sensors on a board) (2) DWT analysis of each obtained signal (3) Calculation of indicator γDWT (4) Classification of cutting tool wear based on indicator γDWT and depth of cut using FFNN. The proposed method is effective for the automatic classification of tool wear conditions.

In [34], the automatic detection of BRB faults in SCIM with soft start is investigated. The detection is tested with four different soft starters. The detection is based on current and stray flux signals. The proposed method for automatic detection consists of the following steps: (1) acquiring current and stray flux signals, (2) applying STFT, (3) dividing the STFT map into a grid of m rows by n columns and calculating the proposed indicator for each region of the map, (4) feature reduction by applying LDA, (5) automatic classification based on FFNN. With the proposed method, automatic detection of BRB faults during soft start is possible. An overall efficiency of 94.4% is achieved.

In [35], the automatic detection of BRB faults in SCIM with soft start is investigated. The detection is tested with four different soft starters. The detection is based on stray flux signals obtained from the air coil in the radial–axial position (P1). The proposed method for automatic detection consists of the following steps: (1) acquisition of the transient stray flux signal, (2) addition of white Gaussian noise to the signal, (3) application of the persistence spectrum method, (4) adaptation of the images, (5) application of the convolutional neural network. An accuracy rate of 99.89% was achieved.

In [36], the author proposed a method for detecting multiple faults in IM under periodic low-frequency fluctuating loads. In the study, the following conditions are investigated individually: healthy, partially broken bar, one broken bar, eccentricity due to unbalance, and eccentricity due to misalignment. The proposed method consists of the following steps: (1) data acquisition, (2) feature extraction (time domain), (3) application of Self-Organizing Maps, (4) feature reduction by linear discriminant analysis, (5) application of a neural network classifier. A triaxial sensor was used for data acquisition. The sensor itself is fixed to the frame and consists of Hall-effect transducers that measure axial, radial, and axial–radial flux. The authors report global classification rates of approximately 99.5% and 99% during training and testing, respectively.

Table 1 summarizes the articles from this section.

## 3. Materials and Methods

**Figure 3:**
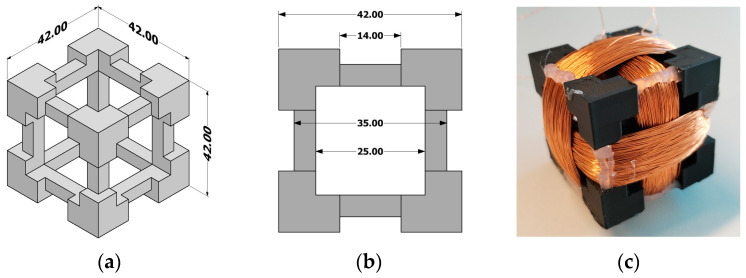
(**a**) Technical drawing of the sensor body; (**b**) cross-section of the sensor body; (**c**) photograph of the finished triaxial.

The test setup consists of a triaxial sensor, a data logger, a laptop, a servo machine test system, and test objects. The triaxial coil consists of three copper coils that are perpendicular to each other. Each coil was wound by hand and had 500 turns. The nominal diameter of the single-coated enameled wire was 0.2 mm (class 200, grade 1). The body for the triaxial coils was drawn in Autodesk Inventor Professional and 3D printed using UltiMaker S5. The material used for the body was PLA NX2 (φ 2.85 mm, fill density 100%). Resistance and inductance of each coil were measured with HAMEG Milliohm-Meter HM 8014 and HAMEG LC-Meter 8018. The results are as follows: Coil 1: 43.3 Ω, 14.5 mH, Coil 2: 42.0 Ω, 13.3 mH, Coil 3: 44.1 Ω, 15.5 mH. The model with the dimensions of the body and the finished triaxial sensor is shown in Figure 3.

The data logger used was National Instruments, model USB-6003, and the laptop model was Acer Aspire 5 (11th Gen Intel(R) Core (TM) i5-1135G7 @ 2.40 GHz; 2.42 GHz. Data was recorded using the MATLAB (R2020b) Analog Input Recorder application. The duration of measurement was set to 20 s, and the sampling frequency to 5 kHz. Three channels were used: ai0, ai1, and ai2. After setting the sampling frequency and selecting three channels, the application automatically sets the sampling frequency to 5.3125 kHz. The displayed number of samples per measurement is 100,006.

The servo machine test system from the manufacturer Lucas-Nuelle consists of the control module and the servo motor. The test system is of type CO3636-6V. The servomotor was used as a brake, and the control module was used to set the load torque to a constant value of 3.33 Nm for each measurement.

The test objects were two three-phase, totally enclosed, fan-cooled (TEFC) squirrel cage IM with the same characteristics as shown in Table 2. All IMs were line-fed.

**Figure 4:**
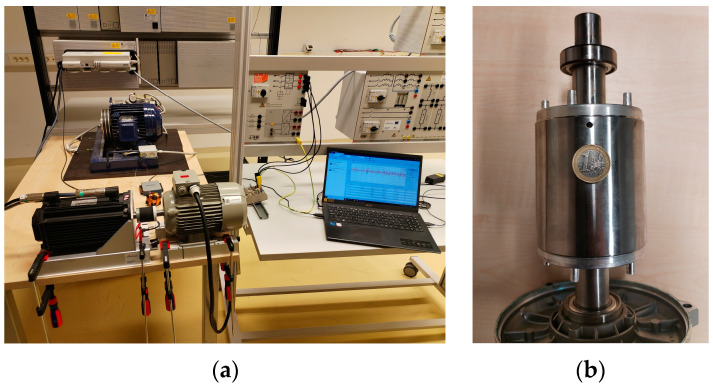
(**a**) Experimental setup; (**b**) simulation of one broken rotor bar—4 mm hole.

The servomotor with its stand was attached to the table with two clamps and was not removed from its fixed position during the entire measurement process. The induction motors were mounted on the hollow metal supports and fixed to the table with three clamps. When the first IM was placed, the position of the beams was marked on the table. The height was leveled by placing a spirit level on the coupling between the servo motor and the IM. The test setup is shown in Figure 4a.

The fault of a broken rotor was simulated by drilling a 4 mm diameter hole in a rotor bar. The generated fault is shown in Figure 4b.

The main idea of the measurement method was the randomness of the positioning of the triaxial sensor on the IM. The randomness includes the random position and the random orientation of the sensor itself. Since randomness can be understood in different ways, the authors agreed on four guidelines before the measurement: (1) at least one vertex of the sensor must be in contact with the IM, i.e., the sensor must always be in contact with the IM; (2) divide the IM into 5 areas—left ribs, right ribs and upper ribs, drive end and non-drive end (plastic fan cap); (3) change the area after each measurement; (4) position the sensor randomly in the given area.

**Figure 5:**
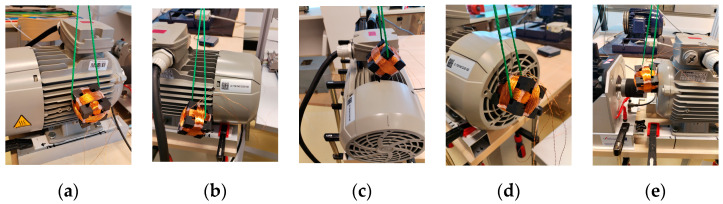
Examples of random positions: (**a**) left ribs; (**b**) right ribs; (**c**) top ribs; (**d**) non-drive end (plastic fan cap); (**e**) drive end.

An example of the measurement in each area can be found in Figure 5. The green rope seen in Figure 5 is used to prevent the hands from touching the coils. During the measurement, there were no observable position changes in the sensor due to the vibration of the motor.

The physical quantity measured is the induced electromotive force (emf) of each coil. All measurements were taken when the motors had reached a steady state under a load of 3.33 Nm. The measurements were performed on two IMs in the following way: 100 measurements per day with a duration of 10 days for each motor and each state of the IM2; a total of 1000 measurements on the healthy motor, denoted IM1; 1000 measurements on the IM2 in the healthy state, denoted IM2_H, and 1000 measurements on the IM2 in the broken rotor bar state, denoted IM2_BRB1. The measurement interval of 10 days was chosen by the authors to simulate daily or periodic monitoring of the health status of the motor.

The method for detecting a broken rotor bar consists of a statistical analysis of the raw data. The first step is to determine whether the data follows a parametric or non-parametric distribution. The second step is to apply an appropriate statistical test to each set of measurement data [37]. For example, each measurement set of motor IM1 is compared with every other set of motor IM1. The same applies to IM2_H and IM2_BRB1. The detection of a broken rotor bar is only performed for motor IM2. Motor IM1 was kept in a healthy state throughout the investigation to determine if there is a statistically significant difference between two healthy states of different motors, in this case, IM1 and IM2_H.

**Figure 6:**
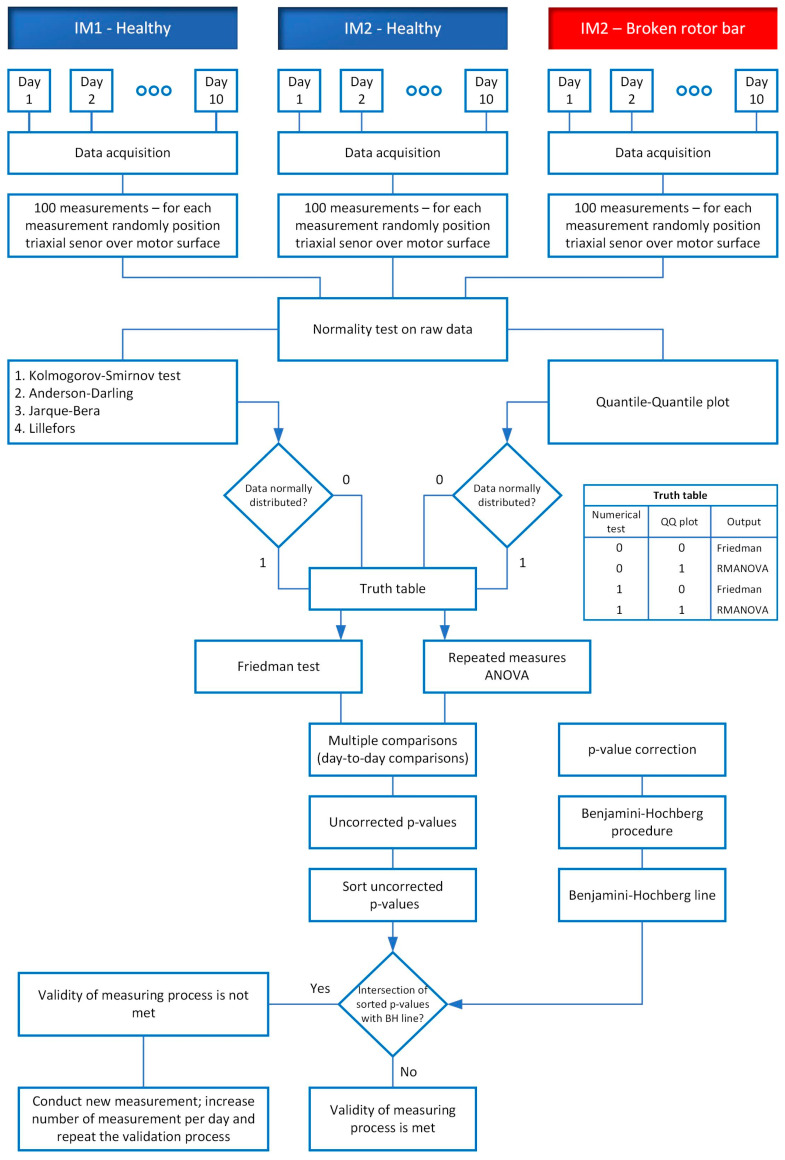
Flow chart of the validation process.

To comprehend more clearly the overall measurement and validation process flow chart is shown in Figure 6. The details of the flow chart are presented in Section 4, Section 5 and Section 6 of this paper.

## 4. Data Analysis

**Figure 7:**
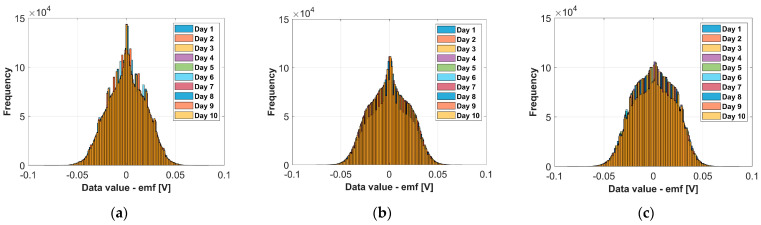
Raw data presentation using histograms: (**a**) IM1-healthy; (**b**) IM2_H-healthy; (**c**) IM2_BRB1-faulty; Number of bins for all histograms is 100.

The data visualization for each motor, each motor state and each day is shown with histograms in Figure 7. The histogram of each day contains all data values obtained with the data logger for all three coils. The number of bins chosen to represent the histogram is 100. This number of bins was chosen for visualization purposes only, i.e., to show 10 histograms in one figure that can be visually distinguished. The visualization is not intended to draw conclusions about the data distribution. The abscissa in Figure 7 represents the emf of the coils.

To determine the statistical method for daily data comparison, i.e., which tests to use, parametric or non-parametric, the data must be examined for normality. There are two approaches to data testing, numerical tests, and graphical interpretation [38]. As stated in [39], there are 55 tests for normality, but in this study, only the tests implemented in MATLAB R2020b are used, i.e., One-sample Kolmogorov–Smirnov, Anderson–Darling, and Jarque–Bera and Lilliefors.

When dealing with a large number of samples, normality tests may detect minimal deviations from normality as significant. Therefore, graphical methods can be a helpful tool for normality decisions [38]. In this paper, a graphical method, the quantile-quantile (Q-Q) plot, is used. The results of the normality tests obtained from MATLAB R2020b are shown in Table 3.

**Figure 8:**
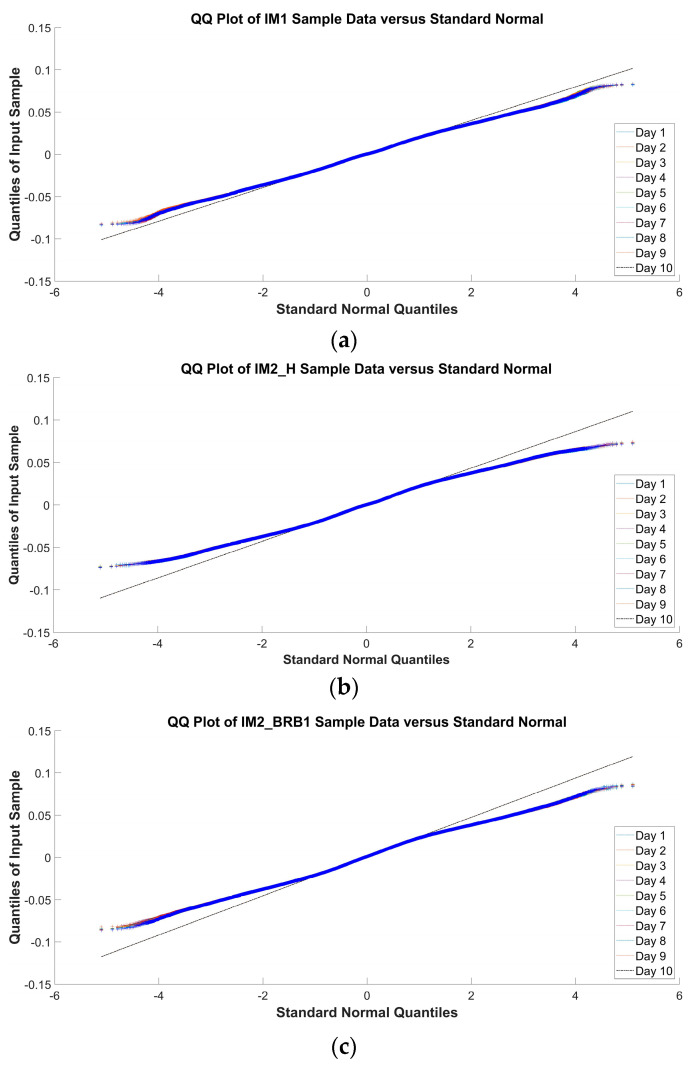
Q-Q plot for (**a**) IM1-healthy; (**b**) IM2_H; (**c**) IM2_BRB1.

The results from Table 3 show that the *p*-value for every motor–day combination is less than 0.001, which means the rejection of the null hypothesis that the data come from a normal distribution. The Q-Q plot for each motor, motor state, and day is shown in Figure 8.

The interpretation of the Q-Q plots in Figure 8 is as follows: The Q-Q plot in Figure 8a shows deviations from the normality line, but not to the extent that suggests non-normal data, leading the authors to conclude that the data from IM1 are subject to a normal distribution; the Q-Q plots in Figure 8b,c show deviations from the normality line, leading the authors to conclude that the data from IM2_H and IM2_BRB1 are subject to non-normal distribution.

The inconsistency between the results from Table 3 and the visual interpretation of the Q-Q plot in Figure 8a, due to both the large sample size and the awareness that the interpretation of Q-Q plots can be subjective [40], led to the decision to apply parametric and non-parametric tests for the daily comparison of the data and the distinction between healthy state and broken bar state.

## 5. Results

### 5.1. Normality Assumption

Because the measurements were conducted on SCIMs over 10 consecutive days (100 measurements per day), meaning that measurements were repeated on the same objects at more than two time points, repeated measures analysis of variance (RMANOVA) as a method for determining the independence of the daily measurements was chosen. RMANOVA is a statistical method used when differences between three or more correlated groups are investigated [41]. The assumptions of the RMANOVA are approximately normally distributed dependent variable, no outliers in any of the repeated measurements, and sphericity [42]. The studies conducted in [43] have shown that RMANOVA is a valid statistical method even in the case of non-normal distribution if the sphericity assumption is met. The sphericity of the data is examined using the Mauchly test [44]. The results of the Mauchly test for each motor and motor condition are shown in Table 4. The Mauchly test is performed with the implemented MATLAB functions “rm = fitrm()” and “mauchly(rm)”.

Statement of the null and alternative hypothesis for the daily measurement of each motor and motor condition: $H_{0}:\mu_{1}=\mu_{2}=\ldots =\mu_{10}$; $H_{1}:$ *at least one measurement* μ *differs from another*.

The results from Table 4 show that the *p*-value for each motor is greater than 0.05, which means that the differences in all daily combinations have equal variances, i.e., the sphericity assumption is met. The results of the RMANOVA analysis are shown in Table 5. The RMANOVA is performed with the implemented MATLAB function “ranova(rm)”.

**Figure 9:**
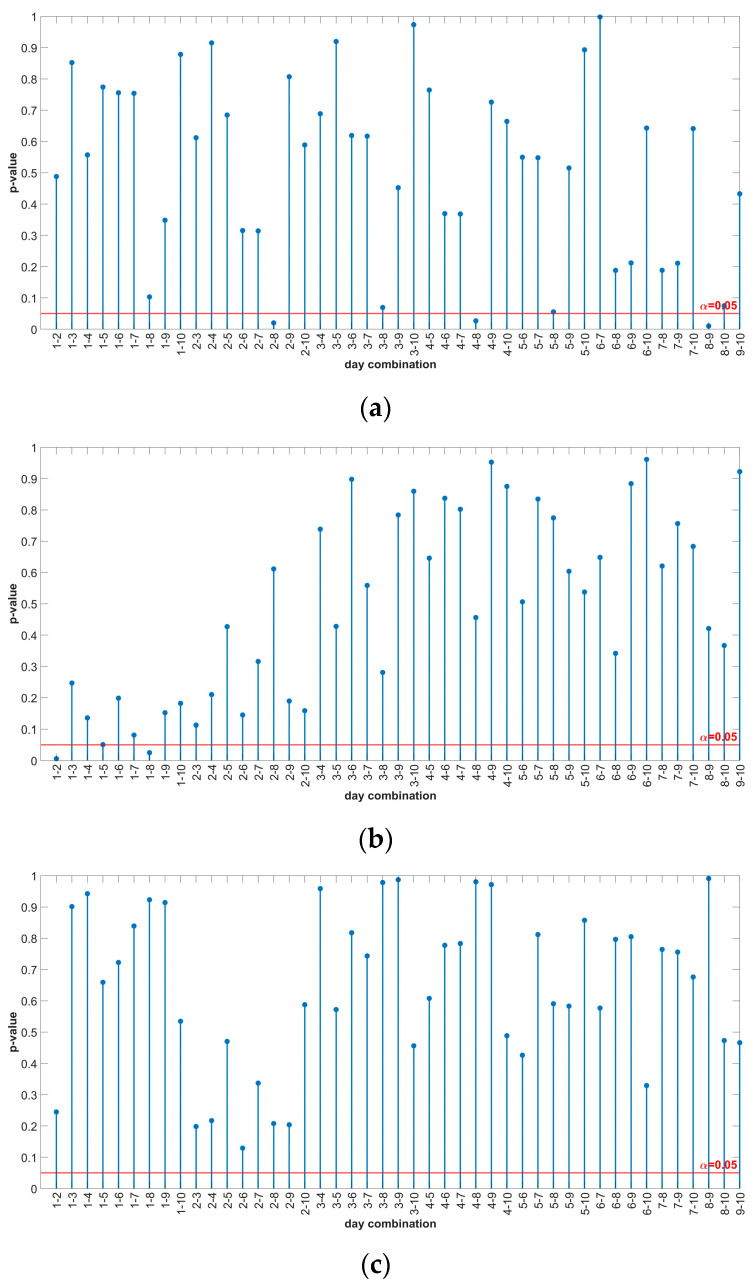
Multiple comparison results of RMANOVA—uncorrected *p*-values: (**a**) IM1-healthy; (**b**) IM2_H-healthy; (**c**) IM2_BRB1-faulty.

The results from Table 5 show that the *p*-value for each motor is greater than 0.05, which means that there is not enough evidence to reject the null hypothesis at a 5% significance level, i.e., all mean values of the 10-day measurements are the same for a given motor. The *p*-values for the daily comparison are determined using the MATLAB function “multcompare(rm)”. The results of the multiple comparison with uncorrected *p*-values are shown in Figure 9.

Figure 9 shows that not all daily combinations have *p*-values of more than 0.05. In Figure 9a,b, the daily combinations with a *p*-value of less than 0.05 are 2-8, 4-8, 8-9, 1-2, and 1-8 (the daily combination 1-5 in Figure 9b has a value of 0.0509).

For multiple hypothesis tests, the probability that the null hypothesis is rejected, even though it is true, increases with the number of tests (Type I error—false positive) [45]. The Type I error is controlled by adjusting the *p*-value. Two general methods for *p*-value adjustment are the familywise error rate (FWER) and the false discovery rate (FDR) [46]. The FWER is the probability of one or more type I errors occurring in a family of tests under the null hypothesis, and the FDR is the expected proportion of the ratio: number of false-positive tests to the number of tests with the null hypothesis rejected [47]. There are a variety of methods for controlling FWER (Bonferroni, Holm, Hochberg, Hommel, and adaptive Bonferroni) and FDR (two-stage linear set-up procedure of Benjamini, Krieger and Yekutieli, Benjamini and Hochberg, and Storey Tibshirani) [48,49,50]. The most representative methods for FWER and FDR are Bonferroni and Benjamini–Hochberg, respectively. Since the Bonferroni correction is conservative and less powerful compared to the Benjamini–Hochberg (BH) correction [51], *p*-value adjustment for the multiple comparison results is performed using the BH correction.

**Figure 10:**
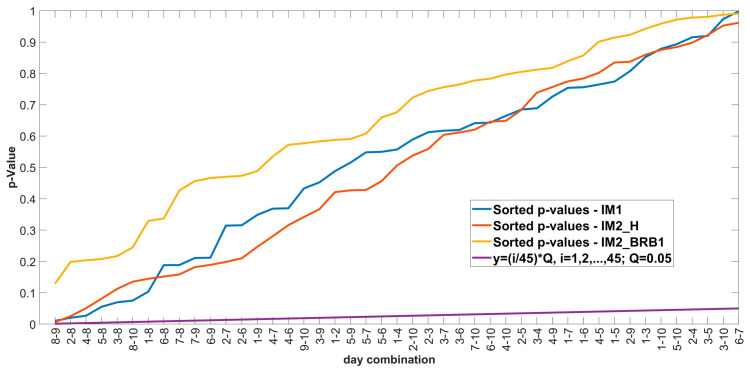
Results of Benjamini-Hochberg procedure.

BH correction method: Sorting the *p*-values in ascending order, ranking the *p*-values (the smallest *p*-value has rank 1), calculating the critical BH value for each *p*-value using the formula (*i*/*m*)*Q*, where *i* is the rank of a particular *p*-value, *m* is the total number of tests, and *Q* is the false discovery rate chosen by the user. After sorting the *p*-values and calculating the critical value, the largest *p*-value whose value is less than the critical value sets the cut-off for rejecting the null hypothesis. All null hypotheses with *p*-values smaller than the largest *p*-value found, including the largest *p*-value, are rejected [52,53]. The results of the BH correction are shown in Figure 10.

The results from Figure 10 show that there is no intersection of the sorted *p*-values with the line *y =* (*i*/*m*)*Q*, which means that all daily combinations with *p*-values below 0.05 are false positives, i.e., there is no statistically significant difference between all daily combinations for each motor.

To investigate whether the approach with a triaxial sensor, random positions over the motors, and raw data make a difference between the motor conditions, a two-way RMANOVA was used. The data for the analysis are organized in a table consisting of columns representing the “day” variable and rows representing the “Motor” variable (IM1, IM2_H, and IM2_BRB1, respectively). The two-way RMANOVA is performed using the MATLAB function “ranova(rm)”. Before performing the two-way RMANOVA, the Mauchly test is performed to check whether the sphericity is met. The result of the Mauchly test is shown in Table 6, and the result of the analysis is shown in Table 7.

**Figure 11:**
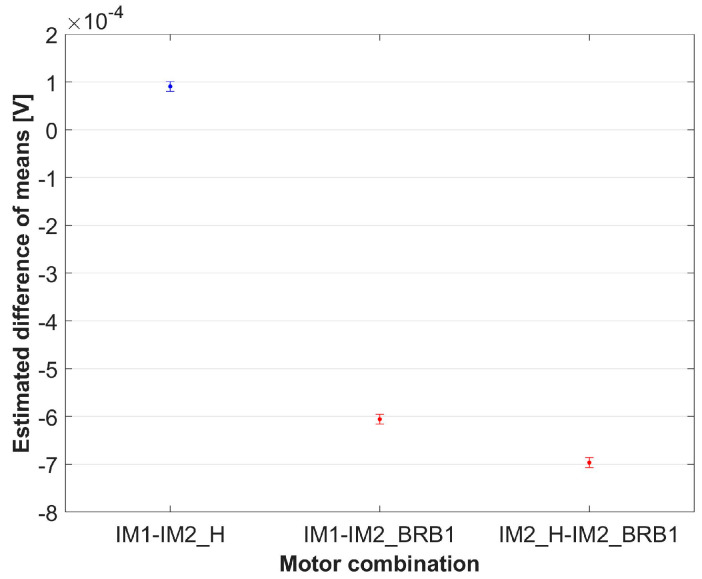
Estimated difference in means of variable “Motor” with 95% confidence interval.

The results from Table 6 show that sphericity is met (*p* > 0.05). The results from Table 7 show that there is not enough evidence for the variable “day” to reject the null hypothesis with a significance level of 5%, i.e., there is no significant difference in the mean values between the days. This is already evident in the analysis, which was carried out separately for each motor. Table 7 also shows that there is strong evidence (*p* = 0) for the variable “Motor” to reject the null hypothesis at a significance level of 5%, i.e., there is a statistically significant difference between the motors. The multiple comparison test according to the variable “Motor” is shown in Table 8, and the estimated difference in means (with 95% confidence intervals) is shown in Figure 11.

The results from Table 8 show that there is a statistically significant difference between the individual motors. Figure 11 shows the graphical representation of the results from Table 8. The estimated difference in means with a 95 % confidence interval for healthy–healthy motor combinations is represented in blue color, and healthy–BRB combinations are represented in red color in Figure 11. The non-overlapping intervals of healthy combination with any healthy–BRB combinations graphically indicate a statistically significant difference. From Figure 11, it is observable that healthy–BRB combinations differ significantly in value from healthy–healthy combinations. To quantify the differences, the percentage difference between values of estimated differences in means is calculated, and the results are shown in Table 9.

### 5.2. Non-Parametric Approach

The assumption of a non-normal data distribution implies the use of a non-parametric test. In non-parametric analysis, the data are transformed into ranks or signs [54]. The power of non-parametric tests is generally lower than that of parametric tests, but they are more powerful for non-normally distributed data [54,55]. The non-parametric alternative to the RMANOVA test is the Friedman test [56]. The null hypothesis of the Friedman test states that the compared groups come from the same population or the population with the same median [57]. The null hypothesis for this study is that the data obtained from the measurements taken over a 10-day period for a particular motor come from the same population. The test is performed using the MATLAB function “friedman(x)”, where “x” represents the table of measurements whose columns are assigned to days. The results of the Friedman test for each motor are shown in Table 10.

**Figure 12:**
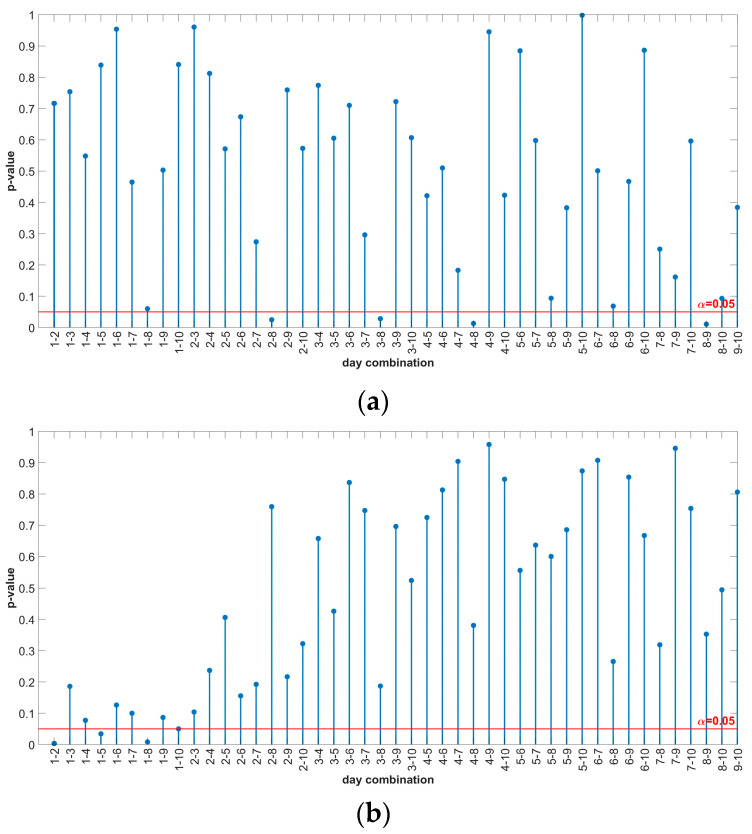

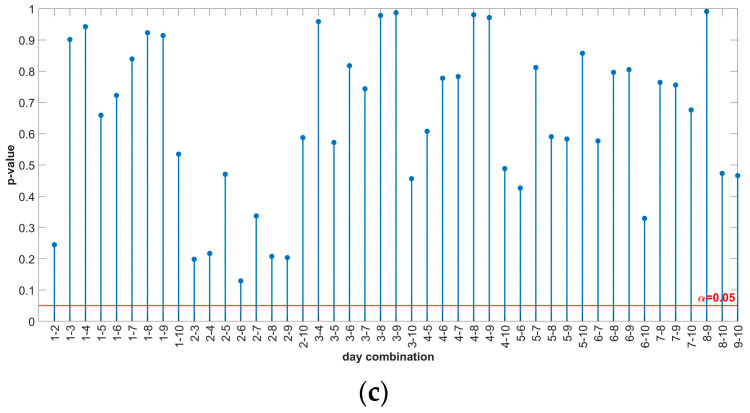
Multiple comparison results of Friedman test—uncorrected *p*-values: (**a**) IM1-healthy; (**b**) IM2_H-healthy; (**c**) IM2_BRB1-faulty.

The results from Table 10 show that all *p*-values are greater than 0.05, i.e., for each motor/motor condition, there is insufficient evidence to reject the null hypothesis at a 5% significance level, meaning that all measurements for a given motor come from the same distribution. The results of the multiple comparison with uncorrected *p*-values for each motor are shown in Figure 12.

**Figure 13:**
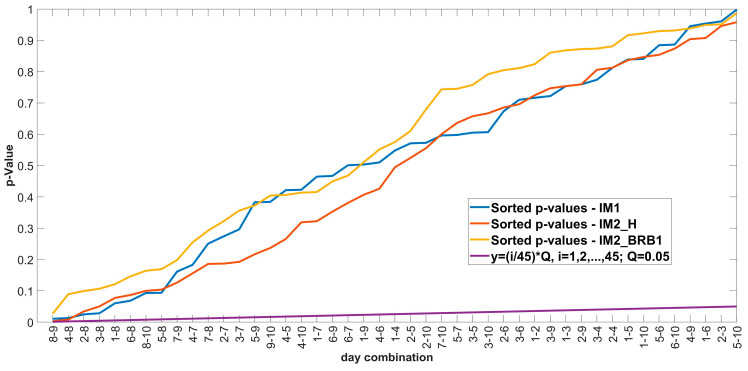
Correction of *p*-values with BH after multiple comparison of the Friedman test: (a) IM1-Healthy; (b) IM2_H; (c) IM2_BRB1.

Figure 12 shows that not all *p*-values are above the significance level of 0.05. To check whether the significant *p*-values are false-positive, the BH correction is applied. The results of the BH correction are shown in Figure 13.

The results from Figure 13 show that there is no intersection of the sorted *p*-values with the line *y* = (*i*/*m*)*Q*, which means that all daily combinations with *p*-values below 0.05 are false positives, i.e., there is no statistically significant difference between all daily combinations for each motor.

To examine the differentiation between motor conditions assuming a non-normal distribution, the Friedman test was used. The data for the analysis are organized in a table consisting of columns representing the variable “Motor” and rows representing the variable “day”. The null hypothesis for this analysis is as follows: the data obtained for each motor with the measurements over a 10-day period come from the same population. The results of the Friedman test are shown in Table 11.

**Figure 14:**
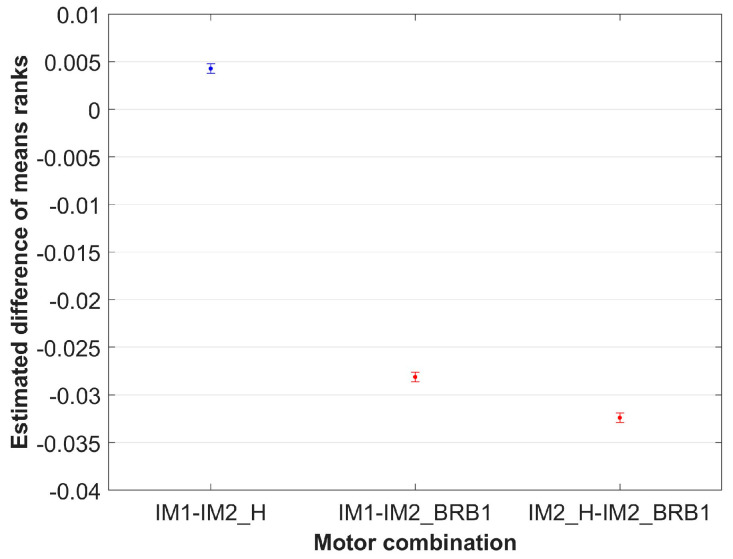
Estimated difference in mean ranks of variable “Motor” with 95% confidence interval.

The results from Table 11 show that there is a significant difference between the motors (*p* = 0), i.e., that the data obtained for each motor does not come from the same population. The multiple comparison by the variable “Motor” is shown in Table 12 and the estimated difference in mean ranks (with 95% confidence intervals) is shown in Figure 14.

The results from Table 12 show that there is a statistically significant difference between the individual motors. Figure 14 shows the graphical representation of the results from Table 12. The percentage difference in estimated mean ranks for the motor combinations with BRB fault compared to the healthy motor combinations is shown in Table 13.

## 6. Conclusions

In this study, a statistical approach for the validation of measurement methods and the detection of broken rotor bars was investigated. A novel approach to detection is the random positioning of the sensor (triaxial coil) over the surface of the induction motor. The study was conducted for two motors and three cases: The first motor was kept in a healthy state throughout the measurement; for the second motor, the measurement was performed in a healthy state, after which a fault, i.e., a broken rotor bar, was generated.

Statistical tests were performed on the raw data, i.e., values of the induced electromotive force of the coils. The data was collected over a period of ten days, with 100 measurements per day. The statistical analysis of the data distribution was inconclusive. The normality tests performed in MATLAB did not show complete agreement with the Q-Q plots. Two approaches were chosen: parametric and non-parametric. RMANOVA was used for the parametric approach. The RMANOVA results to validate the measurement method show that the measurements are time-independent for each motor and motor condition. The multiple comparison analysis was performed together with the BH *p*-value correction for the daily measurement combinations. After applying the BH correction, all *p*-values were above the 5% significance level, i.e., there was no statistically significant difference between the daily measurements for a given motor. For BRB detection, a two-way RMANOVA was used to statistically analyze the differences between motors. The analysis shows that there is a statistically significant difference between all motors/conditions. However, the results between two healthy conditions (IM1 and IM2_H) were compared, and their data were found to be statistically different; the quantitative analysis of the estimated mean difference shows a small difference in the mean values for the healthy motor combination compared to the combination of healthy and BRB motors. All conclusions for the RMANOVA also apply to the non-parametric approach. The only difference is that the Friedman test uses ranks, and therefore, the results for the motor comparison are expressed as estimated differences in the mean ranks.

Overall, the results show that the parametric and non-parametric approaches lead to the same conclusions: the measurement method is valid, i.e., it provides consistent results over time, and the raw data obtained with random positioning of the triaxial sensor can statistically distinguish a healthy motor from a motor with a broken rotor, but the reference point or threshold needs to be specified.

Limitations of this research are the number and type of motors, i.e., the experiments are conducted on two SCIMs of same manufacturer and technical characteristics, severity of the fault, i.e., only case of one broken bar is investigated, time duration and sampling frequency of the measurement was kept constant throughout the experiment, meaning their influence has not been investigated, the influence of the number of measurements per day has not been investigated and the fact that healthy motor as reference is needed to detect broken rotor fault.

Future work will include statistical analysis of rotor faults with two broken bars (adjacent and non-adjacent) and a comparison of data from IMs from different manufacturers to investigate the threshold for the healthy–healthy and healthy–BRB combinations.

## Figures and Tables

**Table 1:** Summary of literature review.

Reference	Sensor Position (Ref. to Figure 2)	Sensor—Type and Dimensions	Fault Detection Method	Analysed Fault	Tested SCIM Rated Power/ Facility/Motor Supply
[19]	P1, P2, P3	Circular coil; N = 1000; Inner φ = 3.9 cm; Outer φ = 8 cm; Height 1 cm	STFT DWT	Misalignment Misal. + 1 BRB Misal. + 2 BRB (adjacent)	M1: 1.1 kW M2: 0.75 kW Lab. Start-up Line supply
[20]	P1	Circular coil; N = 1000 Inner φ = 3.9 cm Outer φ = 8 cm Height 1 cm	STFT FFT	2 BRB (adjacent, half pole pitch and one pole pitch)	M: 1.1 kW Lab. Steady state Line supply
[21]	P1, P2	Circular coil; N = 1000 Inner φ = 3.9 cm Outer φ = 8 cm Height 1 cm	FFT Spectral subtraction Autocorrelation	2 BRB (5 combinations)	M: 1.1 kW Lab. Steady state Line supply
[22]	P1, P2, P3, P4	Circular coil; N = 1000 Inner φ = 3.9 cm Outer φ = 8 cm Height 1 cm	Bispectrum Autocorrelation	1 BRB	M: 1.1 kW Lab. Steady state And Start-up Line supply
[23]	P1	Circular coil Nc =1000 Inner φ = 3.9 cm Outer φ = 8 cm Height 1 cm	STFT	1 BRB 2 BRB (adjacent)	M: 1.1 kW Lab. Steady state Line supply
[24]	P1	Helmholtz coil Nc =320 Inner φ = 121 cm Outer φ = 155 cm	FFT	1 BRB 2 BRB	M1:5.5 kW Lab. Steady state Line supply M2,3: 280 kW, 6.6 kV, Field test Steady state Line supply
[25]	P1, P2, P3	Circular coil Nc =1000 Inner φ = 3.9 cm Outer φ = 8 cm Height 1 cm	MUSIC FFNN	1 BRB 2 BRB	M1: 1.1 kW M2: 7.5 kW Lab. Start-up Line supply
[26]	P1, P2	Triaxial stray flux sensor Three hall sensors perpendicular axis to each other Allegro—A1325	STFT Statistical parameters LDA dimensionality reduction FFNN	Misalignment Misal. + 1 BRB Misal. + 2 BRB (adjacent)	M:0.74 kW Lab. Start-up Line supply
[27]	P1	Circular coil Nc = 320	FFT STFT	1 BRB 2 BRB (adjacent) 2 BRB (non-adjacent; 90° el. apart)	M: 7.5 hp Lab. Start-up and steady state Line supply
[28]	P1	Square body Nc = 1500 copper wire φ = 0.1 mm Inner square length 40 mm Outer square length 50 mm Height 4.5 mm	FFT	1 BRB	M:4 kW Lab. Steady state Line supply
[29]	P1	Circular coil Nc =300 (as stated in text)	FFT	1 BRB 2 BRB adjacent 2 BRB non-adjacent Load unbalance Misalignment Eccentricity	M1:7.5 kW M2: 5.5 kW M3: 2.0 kW M4: 5.5 kW Lab. Steady state Line supply
[30]	P1	Square body Nc = 3500 Inner square length 40 mm Outer square length 50 mm Height 4.5 mm	FFT	Misalignment Eccentricity Bearing fault	M1:750 kW M2: 750 kW M3: 240 kW M4: 240 kW Field testing Steady state Line supply
[31]	P1, P2, P3	Circular coil Nc =1000 Inner φ = 65 mm Outer φ = 80 mm Height 15 mm	FFT STFT	1 BRB 2 BRB (adjacent)	M: 1.1 kW Lab. Start-up and steady state 4 soft-starters
[32]	P1	Triaxial stray flux sensor Three perpendicular hall-effect sensors	STFT FFNN	1 BRB 2 BRB (adjacent) Misalignment	M1: 1 hp M2: 1.47 hp Lab. Start-up Line supply
[33]	P1	Triaxial stray flux sensor Three hall sensors mounted perpendicular on a PCB board	DWT FFNN	Cutting tool wear evaluation	M1: 3.7 kW Line supply
[34]	P1	The text description of the coil does not match the coil presented in the paper	STFT LDA FFNN	1 BRB 2 BRB (adjacent)	M: 1.1 kW Lab. Start-up and steady-state 4 soft-starters
[35]	P1	Circular coil Nc =1000 Inner φ = 6.5 cm Outer φ = 8 cm Height 1.5 cm	Persistence spectrum CNN	1 BRB 2 BRB (adjacent)	M: 1.1 kW Lab. Start-up and steady-state 4 soft-starters
[36]	P1	Triaxial stray flux sensor Three perpendicular hall-effect sensors	Self-Organizing Maps NN	1/2 BRB 1 BRB Unbalance Misalignment	M: 1.5 kW Lab. Fluctuating load VFD supply

**Table 2:** Technical characteristics of IM1 and IM2.

Manufacturer: Siemens; Type: 1AV3082B 1LE10030DB222AB4							
V	Hz	kW	A	PF	RPM	EFF-CL	ETA %
400 Y	50	0.55	1.26	0.78	1440	IE3	80.8

**Table 3:** Results of the normality tests.

**Test**	**Motor**	***p*** **-Value**									
		**Day 1**	**Day 2**	**Day 3**	**Day 4**	**Day 5**	**Day 6**	**Day 7**	**Day 8**	**Day 9**	**Day 10**
One-sample Kolmogorov–Smirnov	IM1	<0.001	<0.001	<0.001	<0.001	<0.001	<0.001	<0.001	<0.001	<0.001	<0.001
	IM2_H	<0.001	<0.001	<0.001	<0.001	<0.001	<0.001	<0.001	<0.001	<0.001	<0.001
	IM2_BRB1	<0.001	<0.001	<0.001	<0.001	<0.001	<0.001	<0.001	<0.001	<0.001	<0.001
Anderson-Darling	IM1	<0.001	<0.001	<0.001	<0.001	<0.001	<0.001	<0.001	<0.001	<0.001	<0.001
	IM2_H	<0.001	<0.001	<0.001	<0.001	<0.001	<0.001	<0.001	<0.001	<0.001	<0.001
	IM2_BRB1	<0.001	<0.001	<0.001	<0.001	<0.001	<0.001	<0.001	<0.001	<0.001	<0.001
Jarque-Bera	IM1	<0.001	<0.001	<0.001	<0.001	<0.001	<0.001	<0.001	<0.001	<0.001	<0.001
	IM2_H	<0.001	<0.001	<0.001	<0.001	<0.001	<0.001	<0.001	<0.001	<0.001	<0.001
	IM2_BRB1	<0.001	<0.001	<0.001	<0.001	<0.001	<0.001	<0.001	<0.001	<0.001	<0.001
Lilliefors	IM1	<0.001	<0.001	<0.001	<0.001	<0.001	<0.001	<0.001	<0.001	<0.001	<0.001
	IM2_H	<0.001	<0.001	<0.001	<0.001	<0.001	<0.001	<0.001	<0.001	<0.001	<0.001
	IM2_BRB1	<0.001	<0.001	<0.001	<0.001	<0.001	<0.001	<0.001	<0.001	<0.001	<0.001

**Table 4:** Results of the Mauchly test for each motor and motor state.

	W	ChiStat	DF	*p*-Value
IM1	0.99999	41.49	44	0.5798
IM2_H	0.99999	33.811	44	0.86671
IM2_BRB1	0.99999	36.867	44	0.76842

**Table 5:** Results of the RMANOVA analysis for each motor separately.

		SumSq	DF	MeanSq	F	*p*-Value
IM1	(Intercept): day	0.0032546	9	0.00036162	1.0344	0.40934
	Error(day)	9439.4	2.7 × 10^7^	0.00034961		
IM2_H	(Intercept): day	0.0037357	9	0.00041508	1.0644	0.38553
	Error(day)	10,529	2.7 × 10^7^	0.00038995		
IM2_BRB1	(Intercept): day	0.0015375	9	0.00017084	0.41369	0.92853
	Error(day)	11,150	2.7 × 10^7^	0.00041296		

**Table 6:** Results of the Mauchly test of data prepared for two-way RMANOVA.

W	ChiStat	DF	*p*-Value
1	36.631	44	0.77698

**Table 7:** Results of the two-way RMANOVA.

	SumSq	DF	MeanSq	F	*p*-Value
(Intercept): day	0.0013094	9	0.00014549	0.37871	0.94548
Motor	8.6033	2	4.3017	10413	0
Motor: day	0.0072185	18	0.00040103	1.0439	0.40485
Error(day)	31,118	8.1 × 10^7^	0.00038417	1	0.5

**Table 8:** Results of multiple comparison test by variable “Motor”—uncorrected *p*-values.

Motor 1	Motor 2	Difference	StdErr	*p*-Value	Lower	Upper
IM1	IM2_H	9.0766 × 10^−5^	5.2479 × 10^−6^	5.0809 × 10^−67^	8.048 × 10^−5^	0.00010105
IM1	IM2_BRB1	−0.00060576	5.2479 × 10^−6^	0	−0.00061605	−0.00059548
IM2_H	IM2_BRB1	−0.00069653	5.2479 × 10^−6^	5.0809 × 10^−67^	−0.00070681	−0.00068624

**Table 9:** Percentage difference in estimated differences in means relative to the healthy–healthy motor combination.

Reference	Motor Combination	Percentage Difference in Estimated Differences in Means
IM1–IM2_H	IM1–IM2_BRB1	767.40%
	IM2_H–IM2_BRB1	867.40%

**Table 10:** Results of the Friedman test for each motor separately.

		SS	df	MS	Chi-sq	Prob>Chi-sq
IM1	Columns	92.7364	9	10.304	10.13	0.34
	Error	247,166,451.7636	26,999,991	9.1543		
	Total	247,166,544.5	29,999,999			
IM2_H	Columns	104.678	9	11.6309	11.43	0.2471
	Error	247,189,783.322	26,999,991	9.1552		
	Total	247,189,888	29,999,999			
IM2_BRB1	Columns	62.6033	9	6.95593	6.84	0.654
	Error	247,200,721.8967	26,999,991	9.15559		
	Total	247,200,784.5	29,999,999			

**Table 11:** Results of the Friedman for motor comparison.

	SS	df	MS	Chi-sq	Prob>Chi-sq
Columns	1.8583 × 10^4^	2	9.2914 × 10^3^	1.8647 × 10^4^	0
Error	5.9776 × 10^7^	59,999,998	0.9963		
Total	5.9795 × 10^7^	89,999,999	0.00038417		

**Table 12:** Results of the multiple comparison by variable “Motor”—uncorrected *p*-values.

Motor 1	Motor 2	Difference	*p*-Value	Lower	Upper
IM1	IM2_H	0.0042759	8.3828 × 10^−62^	0.0037707	0.0047811
IM1	IM2_BRB1	−0.028118	0	−0.028623	−0.027613
IM2_H	IM2_BRB1	−0.032394	0	−0.032899	−0.031889

**Table 13:** Percentage difference in estimated differences in mean ranks relative to the healthy–healthy motor combination.

Reference	Motor Combination	Percentage Difference in Estimated Differences in Mean Ranks
IM1–IM2_H	IM1–IM2_BRB1	757.60%
	IM2_H–IM2_BRB1	857.60%

## Data Availability

The data presented in this study are available upon request from the corresponding author.
